# Computed Tomography Derived Coronary Triangulated Orifice Area—Deduction of a New Parameter for Follow-up After Surgical Correction of Anomalous Aortic Origin of Coronary Arteries and Call for Validation

**DOI:** 10.3389/fcvm.2021.668503

**Published:** 2021-06-24

**Authors:** Fleur M. M. Meijer, Philippine Kiès, Diederick B. H. Verheijen, Hubert W. Vliegen, Monique R. M. Jongbloed, Mark G. Hazekamp, Hildo J. Lamb, Anastasia D. Egorova

**Affiliations:** ^1^CAHAL, Center for Congenital Heart Disease Amsterdam Leiden, Leiden, Netherlands; ^2^Department of Cardiology, Leiden University Medical Center, Leiden, Netherlands; ^3^Department of Anatomy and Embryology, Leiden University Medical Center, Leiden, Netherlands; ^4^Department of Cardiothoracic Surgery, Leiden University Medical Center, Leiden, Netherlands; ^5^Department of Radiology, Leiden University Medical Center, Leiden, Netherlands

**Keywords:** computed tomography angiography, surgical correction, coronary triangulated orifice area, clinical outcome, coronary anomaly, anomalous aortic origin of a coronary artery

## Abstract

**Introduction:** Anomalous aortic origin of a coronary artery (AAOCA) from the opposite sinus of Valsalva is a rare congenital abnormality. Computed tomography angiography (CTA) is primarily used as a diagnostic tool to evaluate the anatomy and identify potentially malignant AAOCA variants. Limited data is available on the role of CTA during postoperative follow-up. We aimed to develop an objective CTA derived parameter for diagnostic evaluation and follow-up after surgical correction of AAOCA and correlate the anatomical features to the postoperative outcome.

**Methods:** All consecutive patients who underwent surgical repair of AAOCA from 2001 to 2018 and had pre and postoperative CTA imaging available were included. A retrospective analysis of the pre- and postoperative CTA and the outcomes was performed. The origin and course of the anomalous coronary artery and the ostial dimensions were evaluated and correlated with restenosis of operated coronary artery. To allow an accurate evaluation of the effective orifice area at diagnosis and after surgical repair we deduce and propose a new parameter—the coronary triangulated orifice area (CTOA).

**Results:** Out of the 54 patients who underwent surgical treatment for AAOCA, 11 fulfilled the inclusion criteria. The median follow-up was 19 months [IQR 3;42]. The mean age at surgery was 41 ± 16 years, with six patients (55%) being male. Postoperatively, the angle between the proximal coronary artery and the aortic wall increased from 20 ± 5° to 28 ± 9° (*p* < 0.01) and ostial diameter in the transversal plane increased from 4.1 ± 2.5 mm to 6.2 ± 2.7 mm (*p* < 0.01). The median CTOA increased significantly from 1.6 mm^2^ [IQR 0.9;4.9] to 5.5 mm^2^ [IQR 3;11.8] (*p* < 0.005). During follow-up, in three patients a restenosis of the operated coronary artery was suspected. In these patients, the CTOA only showed a limited postoperative increase of ≤ 1.4 mm^2^.

**Conclusions:** CTA can play an important role in the evaluation of the pre- and postoperative anatomy in AAOCA patients. CTOA may be of use in conjunction with the acute angle take-off and ostial diameter order to comprehensively evaluate the operated ostium after unroofing or patch angioplasty.

## Introduction

Anomalous aortic origin of coronary arteries (AAOCA) from the opposite sinus of Valsalva is a rare congenital abnormality affecting 0.03–0.1% of the population and involving an abnormal origin and course of a coronary artery (1, 2). Depending on anatomical and clinical characteristics, some AAOCA variants are associated with an increased risk of ischemia and sudden cardiac death in children and active young adults and are designated malignant (3, 4). Surgical repair of malignant AAOCA is reported to be safe and effective (4–6). However, data on long term follow-up is currently lacking and there are remaining concerns on identifying the patients at risk of suboptimal surgical outcomes and long-term complications.

In adults, coronary anatomy can be accurately evaluated using computed tomography angiography (CTA) (7, 8) and CTA is the imaging modality of choice to assess the AAOCA origin and course, degree of luminal narrowing, its relationship to surrounding structures and concomitant obstructive coronary artery disease (9). The various pathologic aspects of malignant AAOCA all contribute to a significantly reduced functional ostial area of the anomalous coronary artery causing ischemia and potentially lethal arrhythmias. Limited data is available on the role of CTA during follow-up and the expert consensus on AAOCA does not reflect on the role of CTA in the postoperative setting (10). A number of studies refer to the status of the neo-ostium after surgery (11–15). However, to our knowledge, no objective non-invasive method for measuring and quantifying the functional ostium has been established, and the spectrum of application of CTA in the postoperative setting is yet to be fully explored (11–14). Given the prominent role of surgery in the adequate management of this often young patient group in need of life long surveillance, it is of interest to know how the (neo-) ostial parameters are effected by the surgical interventions and to correlate this with the clinical outcomes (6).

In this study we compared the pre- and postoperative CTA features of a series of patients who underwent surgical correction of malignant AAOCA and deduce a new CTA derived parameter, the coronary triangulated orifice area (CTOA). The origin and course of the anomalous coronary artery and the ostial dimensions were evaluated and correlated with restenosis of operated coronary artery during follow-up.

## Materials and Methods

All consecutive patients (*n* = 54) who underwent surgical repair of AAOCA from the opposite sinus of Valsalva at the Leiden University Medical Center between 2001 to 2018 were reviewed. In that era, postoperative CTA was not a part of routine clinical follow-up and was performed at discretion of the cardiologist. Adolescent and adult patients who had adequate pre- and postoperative CTA imaging available were approached for informed consent and included in further analysis. Patients unable or unwilling to communicate with the research team were excluded. Cases where CTA imaging was of insufficient quality were excluded. Patient data were collected from the electronic medical file system (EPD-Vision®, Leiden) and included: gender, age, comorbidities (a.o. diabetes, hypertension, previous ischemic coronary artery disease), type of AAOCA (originating from left or right sinus of Valsalva), dominancy of the coronary system, presence of symptoms at diagnosis and at follow-up, diagnostic imaging techniques [CTA, coronary angiography or magnetic resonance imaging (MRI)], the surgical technique used (unroofing or ostioplasty), concomitant procedures, adverse cardiac events at follow-up (re-operation or percutaneous coronary intervention (PCI) on the operated coronary artery). The study focused on medium-term outcomes. Therefore, in hospital events in the postoperative setting (<1 month) were excluded.

Patients were scanned using a 64-row CT scanner (Aquillion64, Toshiba Medical Systems, Otawara, Japan; General Electrics LightSpeed VCT, Milwaukee, WI, USA) or with a 320-row CT scanner (Aquilion ONE, Toshiba Medical Systems) using an ECG-triggered protocol. Before scanning patients' heart rate and blood pressure were monitored. In the absence of contraindications, patients with a heart rate exceeding 65 b.p.m. received 50–100 mg oral metoprolol, or 5–10 mg metoprolol intravenously. For optimal heart phase selection, retrospective ECG gating was used. CTA images were reviewed with PACS® software and were reconstructed in multiphase data sets. Datasets were reconstructed from the retrospectively gated raw data with an effective slice thickness of 0.5–0.625 mm using standardized window/ level software CTA settings for vascular structures. Coronary artery anatomy was evaluated using the reconstruction dataset with the least motion artifacts, ranging from the end-systolic phase and mid-to end diastolic phase, depending on the heart rate of the patient.

**Figure 1:**
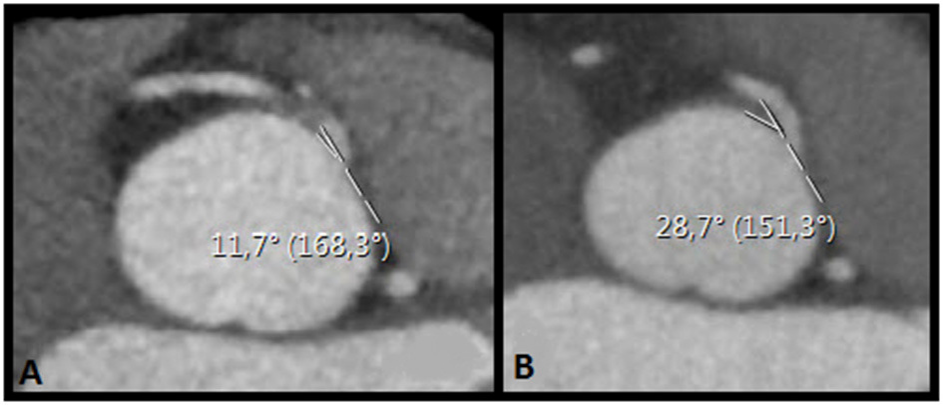
Take-off angles preoperatively **(A)** and postoperatively **(B)** obtained in multiplanar reconstructions in the oblique view at the level of the AAOCA ostium, in the same patient. The angle increased from 11.7 to 28.7°.

**Figure 2:**
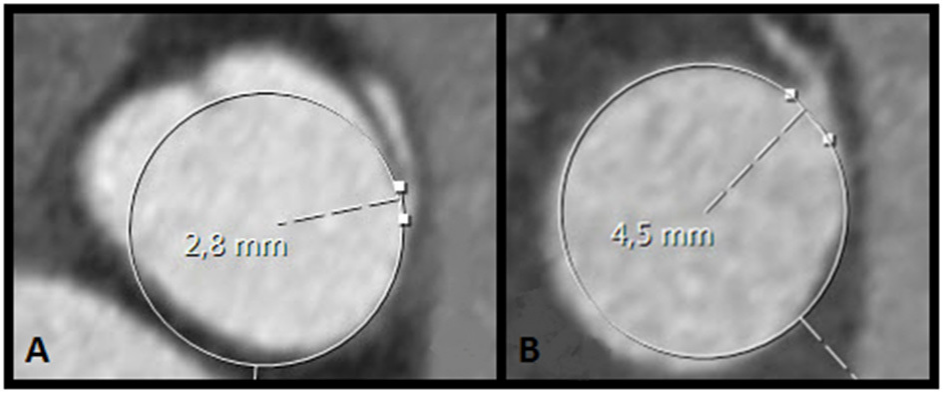
Ostial diameter in the transverse plane preoperatively **(A)** and postoperatively **(B)** obtained in multiplanar reconstructions in the oblique view at the level of the AAOCA ostium, in the same patient. The ostial diameter increased from 2.8 mm to 4.5 mm.

All scans were evaluated by the primary investigator (FMMM), sub-investigator (DBHV) and an experienced radiologist (HJL). Multiplanar reconstructions in the oblique view at the level of the AAOCA ostium using the smallest available slice thickness were obtained and assessed for (1) the presence of an acute angle take-off (Figure 1), defined as the angle between the proximal coronary artery and the tangent line to the aortic wall and (2) the ostial diameter of the AAOCA (Figure 2) (16, 17).

**Figure 3:**
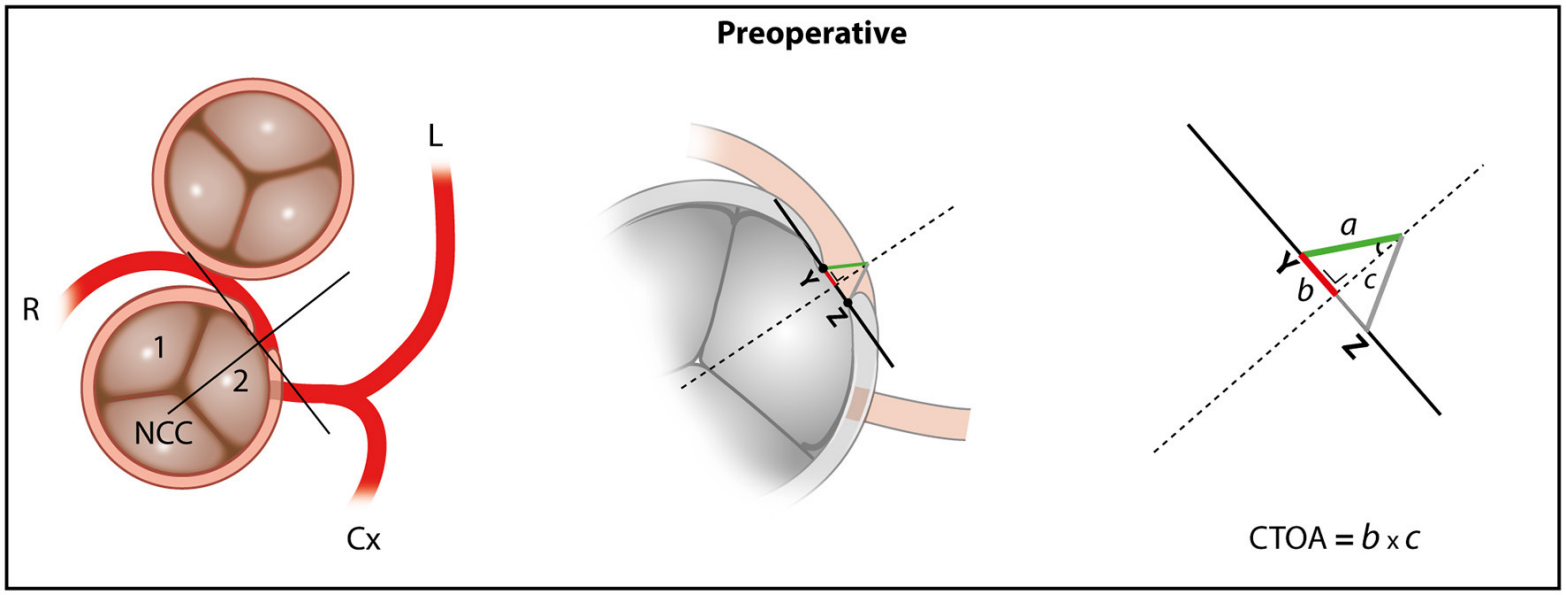
Schematic representation of the coronary triangulated orifice area (CTOA). CTOA = 2 × (½ × b × c) = b × c; b = ½ ostial diameter; c = the depth of the triangle measured on CTA. The effective coronary ostial area as measured by the CTOA increases after the surgical correction of the coronary anomaly. R, right coronary artery; L, left coronary artery; Cx, circumflex coronary artery; NCC, non-coronary cusp; The area of the triangle is formed by 2 equilateral triangles. Y, representing the acute angle the outer edge of the orifice point; Z, representing the end of the ostium of the coronary anomaly; b, the base of the triangle; c, the depth of the triangle.

Since the ostial diameter is a linear parameter and does not take into account the intraluminal depth of the (operated) coronary ostium, it might not fully reflect the functional ostial area of the coronary artery or the benefit attained after surgical correction. This can, in particularly, be the case in patients after surgical patch angioplasty. We therefore deduced the concept of a coronary triangulated ostial area (CTOA) and the methodology of its quantification, which although a 2-dimensional technique, does take into consideration the ostial depth (Figure 3).

**Figure 4:**
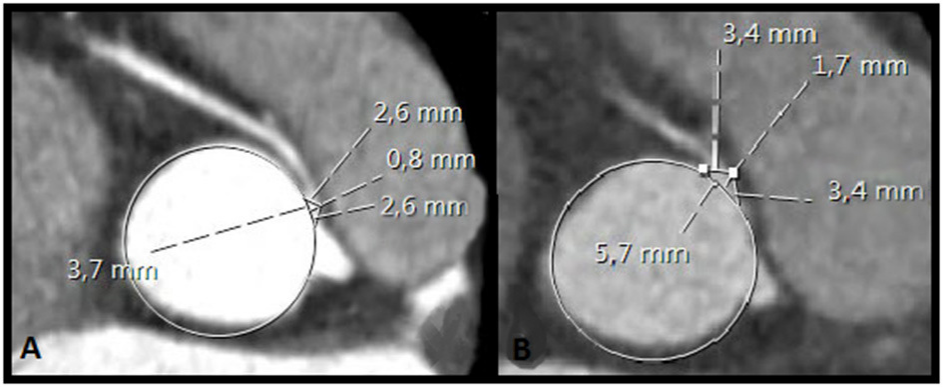
Coronary triangulated orifice area (CTOA) pre- and postoperatively in the same patient (**A,B**, respectively). The ostial diameter increased from 3.7 to 5.7 mm. The CTOA increased from 1.5 to 4.9 mm^2^.

A step by step guide on how to obtain the necessary reconstructions and measure the CTOA can be found in the online supplement (Supplementary Material). In short, a double oblique multiplanar reformation (MPR) reconstruction perpendicular to the aortic valve annulus and parallel to the ascending aorta should be obtained. Next, the investigator scrolls through the double oblique MPR of the aortic root until the center of the orifice of the AAOCA is encountered. A circle is then projected in the orifice of the AAOCA to define where the vessel wall of the AAOCA takes off from the aorta. Next, a tangent line is drawn from the inner edge of the orifice point “Y” to the opposing end of the ostium “Z.” A perpendicular line is placed in the middle of the tangent line which extends to the end of the proximal part of the vessel. Two equilateral triangles are then formed. The area of these two triangles combined is the CTOA. The formula that is used to calculate the area of the ostial triangle is: 2 × (½ x b x c), simplified as b x c. In this formula “b” is the length of the base of the triangle (equal to ½ of the ostial diameter obtained in multiplanar reconstructions in the oblique view at the level of the AAOCA ostium) and “c” the depth of the triangle measured on CTA (Figures 3, 4). To assess the interobserver agreement of CTOA, measurements were repeated by DBHV, blinded to the measurements of FMMM.

### Statistical Analysis

All statistical analyses were performed using IBM SPSS statistics package V.23 (Armonk, New York, USA). Normally distributed continuous data are displayed as mean ± standard deviation (SD) and non-normally distributed continuous data are displayed as median ± interquartile range [IQR1; IQR3]. Proportions are displayed as numbers (percentages, %). For the comparison of values pre- and postoperatively, paired samples *t*-tests or Wilcoxon rank-sum tests were used as appropriate. Interobserver agreement was visually assessed by calculation of the mean difference between observed values and constructing the limits of agreement (±1.96 SD of the difference, thus including 95% of measurements) according to Bland and Altman (18). In addition interobserver agreement was statistically assessed with calculation of intraclass correlation coefficients (ICC). All reported *p-*values were two-sided, and a value of *p* < 0.050 was considered statistically significant.

## Results

**Figure 5:**
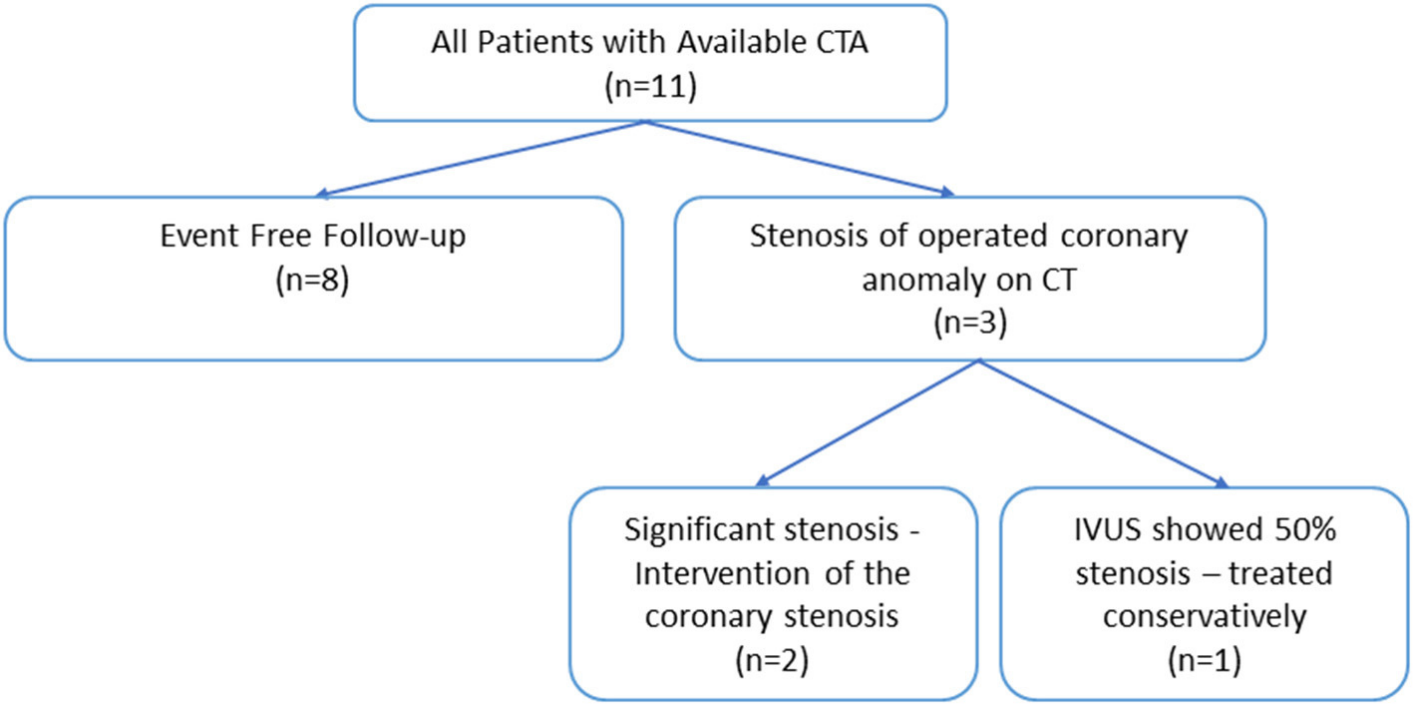
Schematic overview of the study cohort and the outcomes. CTA, computed tomography angiography; IVUS, intravascular ultrasound.

Of the 54 consecutive patients, 11 were included for further analysis (Figure 5). Patient characteristics are shown in Table 1. The mean age at surgery was 41 ± 16 years, with six patients (55%) being male. Preoperatively, one patient had an anomalous left coronary artery LCA (patient 5), all other patients had an anomalous RCA. All patients had an interarterial course with the vessel take-off at or above the pulmonic valve commissure, with the artery traversing between the aorta and the right ventricular outflow tract. Nine patients (82%) had a right coronary dominant system. The majority of the patients underwent coronary artery unroofing (82%). Two patients (18%, patient 5 and 11) underwent an enlargement of the ostium with a saphenous vein patch.

**Table 1:** Baseline characteristics of the patient cohort described in this study.

**Patient characteristics**	**All patients (*n* = 11)**
Male, *n* (%)	6 (55)
Age at surgery, years, mean (SD)	41 (16)
Diabetes mellitus *n* (%)	1 (9)
Hypertension *n* (%)	2 (18)
Previous ischemic coronary artery disease *n* (%)	0
Hypercholesterolemia *n* (%)	3 (27)
AAOLCA *n* (%)	1 (9)
AAORCA *n* (%)	10 (91)
Right dominant system *n* (%)	9 (82)
Symptoms present, *n* (%)	10 (91)
**Primary presentation**, ***n*** **(%)**	
Suspicion of ischemia	8 (73)
Aborted sudden cardiac death	2 (18)
Incidental finding	1 (9)
**Diagnostic imaging techniques**, ***n*** **(%)**	
CTA	11 (100)
CAG	8 (73)
MRI	2 (18)
Interval to follow-up CTA, median months [IQR1; IQR3]	6 (1, 27)
**Indication for follow-up CTA**, ***n*** **(%)**	
Follow-up	4 (36)
Symptoms	
Non-anginal complaints	6 (43)
Typical complaints	1 (9)
**Surgical technique**, ***n*** **(%)**	
Unroofing	9 (82)
Ostioplasty	2 (18)
**Concomitant procedure**, ***n*** **(%)**	
Pulmonary artery patch augmentation	1 (9)

*AAOLCA, anomalous aortic origin of the left coronary artery; AAORCA, anomalous aortic origin of the right coronary artery; CAG, coronary angiography; CTA, computed tomographic angiography; IQR, interquartile range; MRI, magnetic resonance imaging; SD, standard deviation*.

**Figure 6:**
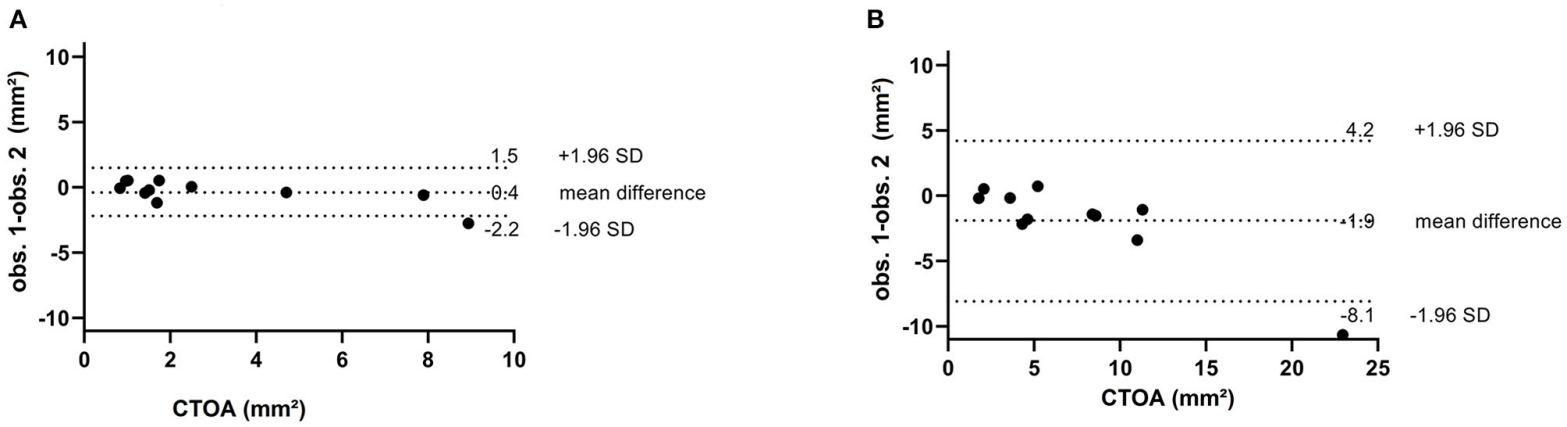
Bland-Altman plots in **(A)** the preoperatively and **(B)** postoperative setting. The mean value of the CTOA is plotted on the x-axis and the difference between the two observers on the y-axis. The mean differences of all observations are close to zero, indicating no important bias between the two observers.

The median interval from operation to CTA was 6 months [IQR1;27]. Figure 5 shows a schematic representation of the CTA findings and consequences and Table 2 shows the patient details. The interobserver variability of the CTOA was evaluated. The intraclass correlation coefficient (ICC) for CTOA is good (ICC = 0.947, *p* < 0.050 for the preoperative measurements and ICC 0.874, *p* < 0.050 for the postoperative measurements). The measurements of both observers are visualized in Bland–Altman plots (Figure 6). This visually confirms that the limits of agreement (dotted lines) of CTOA are acceptable.

**Table 2:** Individual patient characteristics at surgery and follow-up.

**Patient**	**Age at surgery (years)**	**AAOCA**	**Surgical technique**	**Interval to follow-up CTA (months)**	**Indication for follow-up CTA**
1	20–25	RCA	Unroofing	6	Unknown
2	30–35	RCA	Unroofing	<1	Postoperative evaluation
3	56–60	RCA	Unroofing	43	Non-anginal complaints
4	45–50	RCA	Unroofing	2	Non-anginal complaints
5	10–15	LCA	Ostioplasty	<1	Postoperative evaluation
6	25–30	RCA	Unroofing	42	Non-anginal complaints
7	60–65	RCA	Unroofing	13	Typical complaints
8	45–50	RCA	Unroofing	27	Non-anginal complaints
9	55–60	RCA	Unroofing	25	Non-anginal complaints
10	45–50	RCA	Unroofing	1	Non-anginal complaints
11	25–30	RCA	Ostioplasty	2	Non-anginal complaints

*AAOCA, anomalous aortic origin of coronary artery; CTA, computed tomography angiography; LCA, left coronary artery; RCA, right coronary artery*.

A restenosis of the operated coronary artery was suspected in 3/11 patients (27%) based on the CTA (patient 3, 7, and 11). This was further evaluated with coronary artery angiography (CAG) and intravascular ultrasound (IVUS) in all cases. Patient 3 experienced non-anginal complaints and CTA performed 43 months postoperatively suggested an ostial stenosis of the RCA. During CAG a 50% ostial lesion of the RCA was visualized and considered hemodynamically non-significant. The patient was treated conservatively and symptoms resolved. Patient 7 had typical anginal complaints and underwent a CTA that suggested a significant ostial stenosis of the RCA. This was confirmed at CAG with concomitant IVUS evaluation (persistent ostial narrowing after unroofing, visual stenosis of up to 80%, minimum lumen area of proximal RCA 3.3 mm^2^) and the patient underwent a PCI with stent implantation. Patient 11 also underwent a CTA 2 months after surgery due to non-anginal complaints that revealed an ostial lesion of the RCA. A CAG with IVUS assessment was performed and this revealed an ostial stenosis of (60–70%) and a 70% stenosis distally of the venous patch with minimum lumen area of 3.3 and 3.5 mm^2^, respectively. This was treated with PCI with implantation of 2 stents (with overlap).

A retrospective analysis of the pre- and postoperative CTA characteristics was performed, Table 3. The angle between the proximal coronary artery and the aortic wall significantly increased from 20 ± 5° to 28 ± 9° (*p* < 0.01) postoperatively. The ostial diameter, significantly increased from 4.1 ± 2.5 mm to 6.2 ± 2.7 mm postoperatively (*p* < 0.01).

**Table 3:** Pre- and postoperative CTA characteristics.

**CTA Parameters**	**Preoperative (*n* = 11)**	**Postoperative (*n* = 11)**	***P*-value**
Acute angle take-off (°) mean ± SD	20 ± 5	28 ± 9	<0.001
Ostial diameter (mm) mean ± SD	4.1 ± 2.5	6.2 ± 2.7	<0.001
Coronary triangulated orifice area (mm^2^) median [IQR1; IQR3]	1.6 [0.9;4.9]	5.5 [3.7;11.8]	<0.005
No significant stenosis (*n* = 8) [IQR1;IQR3]	2.0 [1.5–7.4]	9.2 [5.4;12.5]	<0.005
≥50% stenosis (*n* = 3) [IQR1;IQR2]	0.9 [0.75;0.85]	1.9 [1.81;1.88]	0.011

*CTA, computed tomography angiography; IQR interquartile range; SD, standard deviation*.

The median CTOA increased significantly from 1.6 mm^2^ [IQR 0.9;4.9] to 5.5 mm^2^ [IQR 3;11.8] (*p* < 0.005). In every patient there was a consistent increase in CTOA compared to preoperative imaging (Table 3). Interestingly, the 3 patients suspected of a restenosis, based on the follow-up CTA all had an only marginal CTOA increase of ≤ 1.4 mm^2^ (Supplementary Table 1).

## Discussion

Computed tomography angiography (CTA) is an important tool for evaluation of the pre- and postoperative anatomy in AAOCA patients. Previously described high-risk findings in coronary artery anomalies include an acute angle take-off of the coronary artery relative to the aorta, an initial aortic intramural course, a “slit like” coronary artery ostium and coursing of the artery between the aorta and the pulmonary trunk. In this study, we compare several objective CTA parameters before and after surgical correction of AAOCA. To allow an accurate evaluation of the effective orifice area we deduce a new parameter—the coronary triangulated orifice area (CTOA). We evaluate the CTOA and correlate the anatomical features to the postoperative outcome in our patient cohort. The main findings of this study are that after surgical correction, the angle between the proximal coronary artery and the aortic wall and the ostial diameter both increase significantly. The CTOA also shows a significant increase postoperatively. Of interest, in patients with a restenosis of the operated coronary artery, the CTOA only showed a very limited postoperative increase.

CTA is currently one of the preferred techniques to evaluate AAOCA. Guidelines provide a Class I recommendation for either CT or magnetic resonance imaging coronary angiography to be used as the initial evaluation method for coronary anomalies as these techniques have the ability to characterize multiple anatomical features of AAOCA (19). The AHA/ACC 2018 or the ESC 2020 guidelines, however, do not give guidance on how to use CTA in the postoperative setting of AAOCA (20). In the literature an acute angle is defined as the angle between the proximal coronary artery and the aortic wall of <45° (21, 22). It is measured as the angle between the plane formed by the ostium center to a point 5 mm along the vessel centerline, and a plane tangent to the aorta in multiplanar axial reconstruction at the level of the AAOCA ostium using the smallest available slice thickness (allowing for highest spatial resolution) (21, 23). In two autopsy studies an acute angle was frequently encountered in patients with AAOCA who presented with sudden cardiac death (24, 25). In our patient cohort we observed a significant increases in the angle postoperatively from 20 ± 5° to 28 ± 9° (*p* < 0.01). According to the current definition, however, the acute angle persisted in all of our patients. This suggests that the finding of an acute angle alone is not reflective of surgical success in a substantial number of patients and cannot as such be predictive of the clinical outcome.

Another parameter used to assess surgical success is shortening of the intramural segment of the AAOCA. Prior research illustrates that there is a high correlation in the use of CTA to identify an intramural course and direct anatomical findings during surgery, and that there is an association with the length of the intramural segment and prognosis of the patients (15, 22, 26). Histologically, an intramural coronary is defined as an coronary artery partly sharing the media of the aortic wall with no adventitia interposed (10, 27). However, this parameter is very hard to accurately delineate on CTA due to the spatial resolution of this technique. For this reason we did not measure the intramural segment in our study. Instead, we opted for measuring the ostial diameter on CTA and calculating the CTOA. This is a geometric derivative of the ostial diameter and ostial luminal depth. In our patient group surgery led to consistent improvement of CTOA from 1.6 mm^2^ [IQR 0.9;4.9] to 5.5 mm^2^ [IQR 3;11.8] (*p* < 0.005), respectively. This parameter may be used to objectify the functional increase in the orifice surface area after surgery. This is particularly applicable in patch augmentation techniques and unroofing in which the ostium is widened. In our study 2 patients underwent PCI of the ostial segment of the operated coronary artery during follow-up. Although the mechanism is not entirely elucidated, ostial restenosis may be caused by fibrous scar tissue formation postoperatively (28). Of interest, in the patients who required PCI during follow-up, the CTOA only showed a very limited postoperative increase of <1.4 mm^2^ to an absolute CTOA of <4mm^2^ (Supplementary Table 1). It would be important to evaluate whether there is an absolute minimal ostial area required for preservation of coronary patency in the postoperative setting and whether a (limited) increase in CTOA has any prognostic value.

The majority of patients with high risk features of an AAOCA undergo unroofing, this was also the case with 9 out of 11 patients in the current series. With unroofing the (inside) aortic wall part of the intramural course is removed, creating an expanded ostium. The aim of this treatment is to discard the slit like orifice, acute angle, as well as the intramural course. An alternative method is enlargement of the ostium using a patch. This was performed in two patients in our study. Since the ostial diameter is a linear parameter and does not take into account the depth of the (operated) coronary ostium which is widened with unroofing and osteoplasty, it might not fully reflect the surgical benefit, the CTOA does take this depth into account. Our recent work showed that adolescents and adults with AAOCA can present with a wide range of complaints, only 35% of them being classified as “typical” according to the current criteria. Although surgical correction leads to a reduction of symptoms in the vast majority of patients, novel anginal complaints after surgery should prompt further evaluation for potential lesions of the operated coronary artery and suboptimal surgical outcomes (29). CTA can play an important role in the initial objective assessment of the postoperative ostium (and systematic comparison of the postoperative result to the preoperative anatomy). One should, however, realize that this is only measured during a fixed phase of the heart cycle, not allowing for the thorough evaluation of the dynamic component of AAOCA. The full spectrum of application of CTA in the postoperative setting is yet to be explored and no objective non-invasive method for measuring and quantifying the ostial area has been established. The current study is one step further to determine the optimal way to non-invasively quantify the coronary orifice area after surgery and correlate this with clinical outcomes and should be technically and clinically validated in the setting of the larger prospective cohort (30).

## Limitations and Future Perspectives

Despite the role of the Leiden University Medical Center as a national referral center for patients with AAOCA, the patient cohort size is small, reflecting the rarity of the condition and the lack of (historical) structural CTA follow-up. Due to the retrospective nature of this study it is subject to inherent bias, including selection bias based on the clinical and anatomical characteristics of patients that were referred for surgery and therefore underwent more extensive preoperative testing and those with postoperative symptoms requiring further analysis and follow-up.

It is important to note that measurement of the CTOA requires strict adherence to a predefined protocol to ensure its reproducibility (particularly when looking to compare the pre- and postoperative anatomy), which may challenge its robust clinical implementation. Also, the accuracy of the CTOA may in practice be limited by the spatial resolution of the CTA. In theory, the highest in-plane spatial resolution achievable approaches 0.4 mm. In practice, coronary artery motion produces both spatial and temporal blurring, resulting in an effective spatial resolution closer to 0.5 mm (31). The current results, however, do provide us with potentially useful conceptual tools to evaluate surgical outcomes in patients with AAOCA and call for further prospective validation in a larger patient group. The potential of artificial intelligence in automated measurements of CTOA remains to be explored.

One should be aware of the inherent limitations of CTA, predominantly useful when evaluating “high risk” anatomical features, but lacking the ability to adequately asses the physiological consequences of an AAOCA. In the setting of AAOCA associated ischemia, future research should also take into account the functional (dynamic) consequences of these anatomical features and correlate CTA findings with functional evaluation at rest and during (physical) stress using a.o. intravascular ultrasound and fractional flow reserve assessment (32). Adrenalin and dobutamine infusions can be used to mimic physical exercise stress (increasing heart rate and stroke volume) according to the previously published protocols (32, 33). Multicenter studies with standardized preoperative and follow-up protocols are essential in this. To this aid a national protocol for evaluation and management of AAOCA patients has recently been initiated as part of the MuSCAT study (30).

## Data Availability Statement

The raw data supporting the conclusions of this article can be made available by the authors, without undue reservation.

## Ethics Statement

All tests and procedures performed involving human participants were in accordance with the ethical standards of the institutional and national guidelines and with the 2013 Helsinki declaration or comparable ethical standards. Appropriate local scientific board approval was obtained for this retrospective medical record study. All patients provided consent for coded registration, analysis and publication of their data.

## Author's Note

Authors take responsibility for all aspects of the reliability and freedom from bias of the data presented and their discussed interpretation.

## Author Contributions

FM, PK, DV, HV, MJ, MH, HL, and AE conceived and designed the analysis, collected the data, contributed data or analysis tool, performed the analysis, and wrote the paper. All authors contributed to the article and approved the submitted version.

## Conflict of Interest

The authors declare that the research was conducted in the absence of any commercial or financial relationships that could be construed as a potential conflict of interest.

## Supplementary Material

Step by Step Guide to measure CTOA.

1. Start in the standard axial plane at the level of the aortic valve annulus (supplementary Figure 1).
2. Perform a double oblique multiplanar reformation (MPR) perpendicular to the aortic valve annulus and parallel to the ascending aorta (supplementary Figure 2).
3. Scroll through the MPR (double oblique, short axis) of the aortic root until the center of the orifice of the AAOCA appears in the plane you are viewing (supplementary Figure 3).
4. Project a circle in the orifice of the AAOCA to define where the vessel wall of the AAOCA takes off from the aorta (supplementary Figure 4).
5. To measure the CTOA, draw a tangent line from the inner edge of the orifice point “Y” to the opposing end of the ostium “Z” (supplementary Figure 5).
6. A perpendicular line is placed in the middle of the tangent line which extends to the end of the proximal part of the vessel. Two equilateral triangles are then formed.
7. The area of these two triangles combined is the CTOA. The formula that is used to calculate the area of the ostial triangle is: 2 × (½ × b × c), simplified as b × c, where “b” is the length of the base of the triangle and “c” the depth of the triangle.

Supplementary Figure 1 Step 1.

Supplementary Figure 2 Step 2.

Supplementary Figure 3 Step 3.

Supplementary Figure 4 Step 4.

Supplementary Figure 5 Step 5–7.
