# Supplementary material for: Computed Tomography Derived Coronary Triangulated Orifice Area—Deduction of a New Parameter for Follow-up After Surgical Correction of Anomalous Aortic Origin of Coronary Arteries and Call for Validation
Source: Front Cardiovasc Med. 2021 Jun 24;8:668503. doi: 10.3389/fcvm.2021.668503 (PMC8263932; doi:10.3389/fcvm.2021.668503)
Supplement: Supplementary file 1 [file Table_1.DOCX]

**Supplementary Table 1. CTA characteristics per individual patient.**

| **Patient** | **Ostial diameter pre-operatively**  **(mm)** | **Ostial diameter post-operatively (mm)** | **Ostial**  **dept**  **pre-operatively** | **Ostial**  **dept**  **post-operatively** | **Delta ostial diameter (mm)** | **CTOA pre-operatively (mm2)** | **CTOA post-operatively (mm2)** | **Delta CTOA (mm2)** | **Post-operative CTA findings** | **CAG and IVUS** |
| --- | --- | --- | --- | --- | --- | --- | --- | --- | --- | --- |
| 1 | 3.3 | 6.5 | 1.5 | 2.8 | 3.2 | 2.5 | 9.1 | 6.6 | n.a.d. | n/a |
| 2 | 2.7 | 5.4 | 1.2 | 2.0 | 2.7 | 1.6 | 5.4 | 3.8 | n.a.d. | n/a |
| 3 | 1.9 | 2.9 | 0.9 | 1.3 | 1.0 | 0.9 | 1.9 | 1.0 | Significant ostial stenosis RCA | Ostium RCA stenosis 50% - treated conservatively |
| 4 | 2.5 | 6.9 | 1.3 | 1.6 | 4.4 | 1.6 | 5.5 | 3.9 | n.a.d. | n/a |
| 5 | 8.6 | 11.1 | 2.4 | 5.1 | 2.5 | 10.3 | 28.3 | 18 | n.a.d. | n/a |
| 6 | 4.9 | 7.7 | 2.0 | 3.3 | 2.8 | 4.9 | 12.7 | 7.8 | n.a.d. | n/a |
| 7 | 3.0 | 3.3 | 0.5 | 1.1 | 0.3 | 0.8 | 1.8 | 1.0 | Significant ostial stenosis RCA | Ostium flattened + treated by PCI + stent |
| 8 | 9.1 | 10.3 | 1.8 | 2.3 | 1.2 | 8.2 | 11.9 | 3.7 | n.a.d. | n/a |
| 9 | 1.8 | 4.8 | 0.8 | 3.9 | 3.0 | 0.8 | 9.4 | 8.6 | n.a.d. | n/a |
| 10 | 3.7 | 5.7 | 0.8 | 1.7 | 2.0 | 1.5 | 4.9 | 3.4 | n.a.d. | n/a |
| 11 | 3.8 | 3.9 | 1.2 | 1.9 | 0.1 | 2.3 | 3.7 | 1.4 | Significant ostial stenosis RCA | Stenosis proximal and distal to the Patch + treated by PCI + stent |

CTOA, coronary triangulated orificel area; n.a.d, no abnormalities detected; n/a, not applicable; CTA, computed tomography angiography; RCA, right coronary artery; CAG, coronary angiography; IVUS, intravascular ultrasound; PCI, percutaneous coronary intervention
