# Supplementary figures and images for: Computed Tomography Derived Coronary Triangulated Orifice Area—Deduction of a New Parameter for Follow-up After Surgical Correction of Anomalous Aortic Origin of Coronary Arteries and Call for Validation
Source: Front Cardiovasc Med. 2021 Jun 24;8:668503. doi: 10.3389/fcvm.2021.668503 (PMC8263932; doi:10.3389/fcvm.2021.668503)

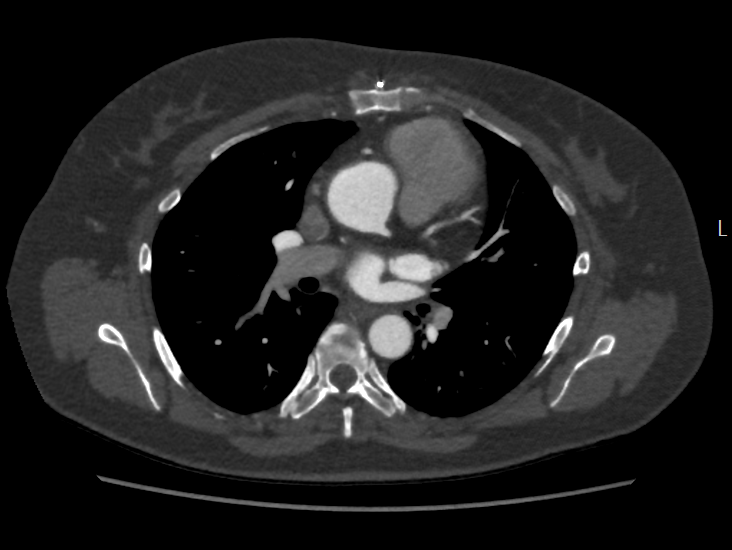

Supplement: Supplementary Figure 1 — Step 1. [file Image_1.TIF]

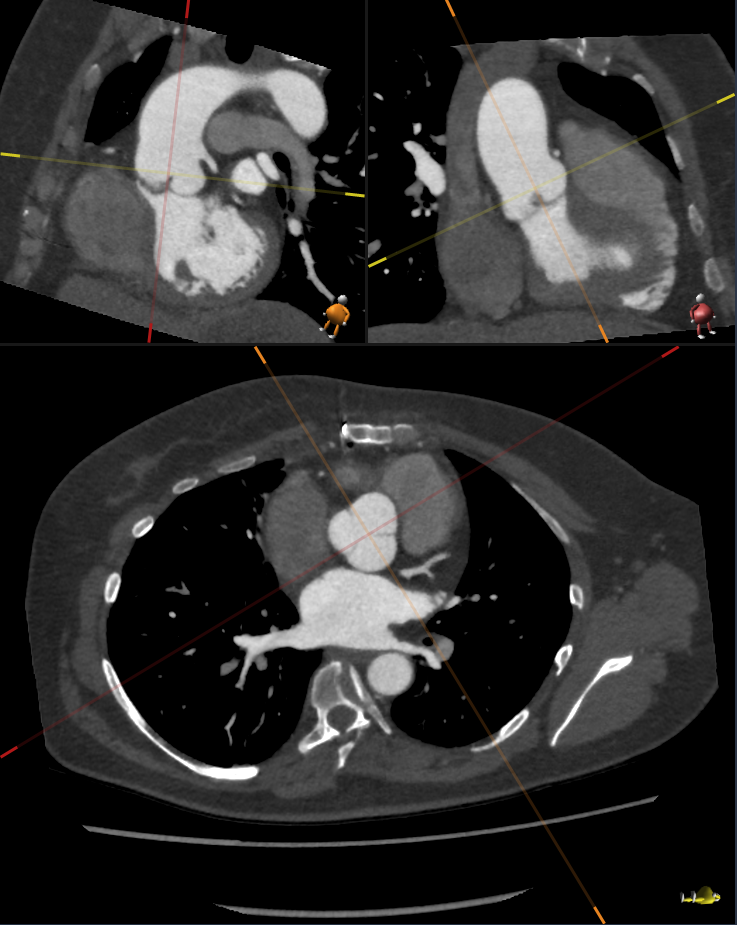

Supplement: Supplementary Figure 2 — Step 2. [file Image_2.TIF]

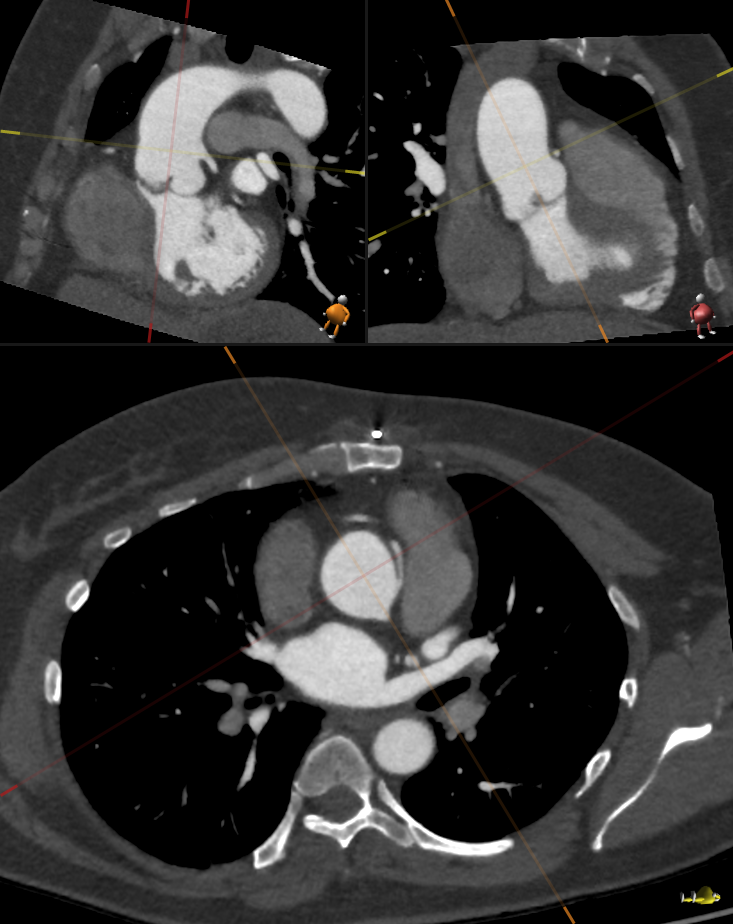

Supplement: Supplementary Figure 3 — Step 3. [file Image_3.TIF]

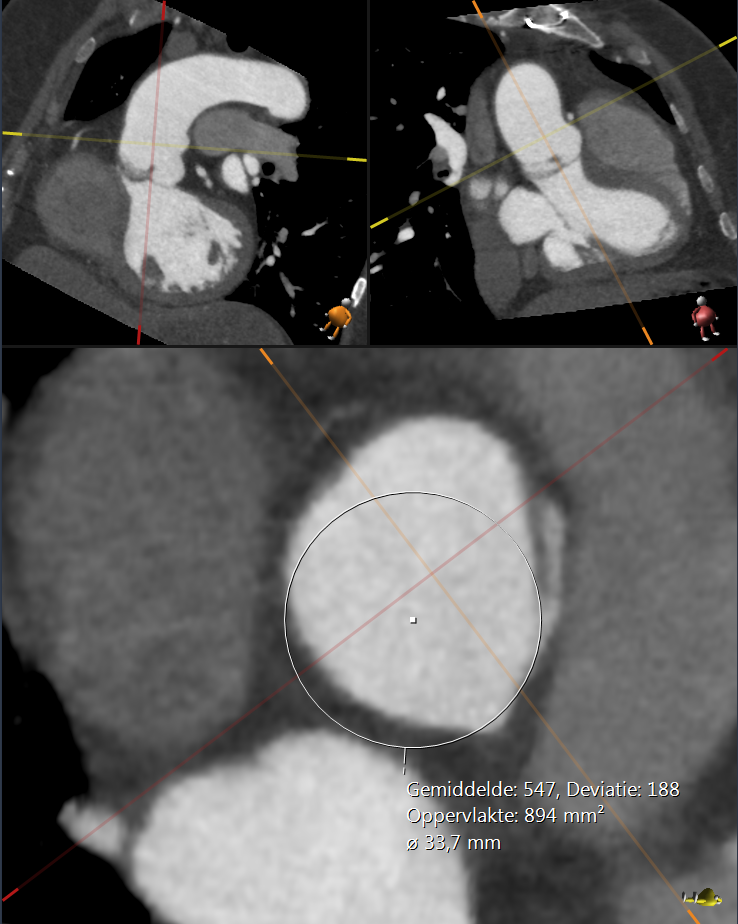

Supplement: Supplementary Figure 4 — Step 4. [file Image_4.TIF]

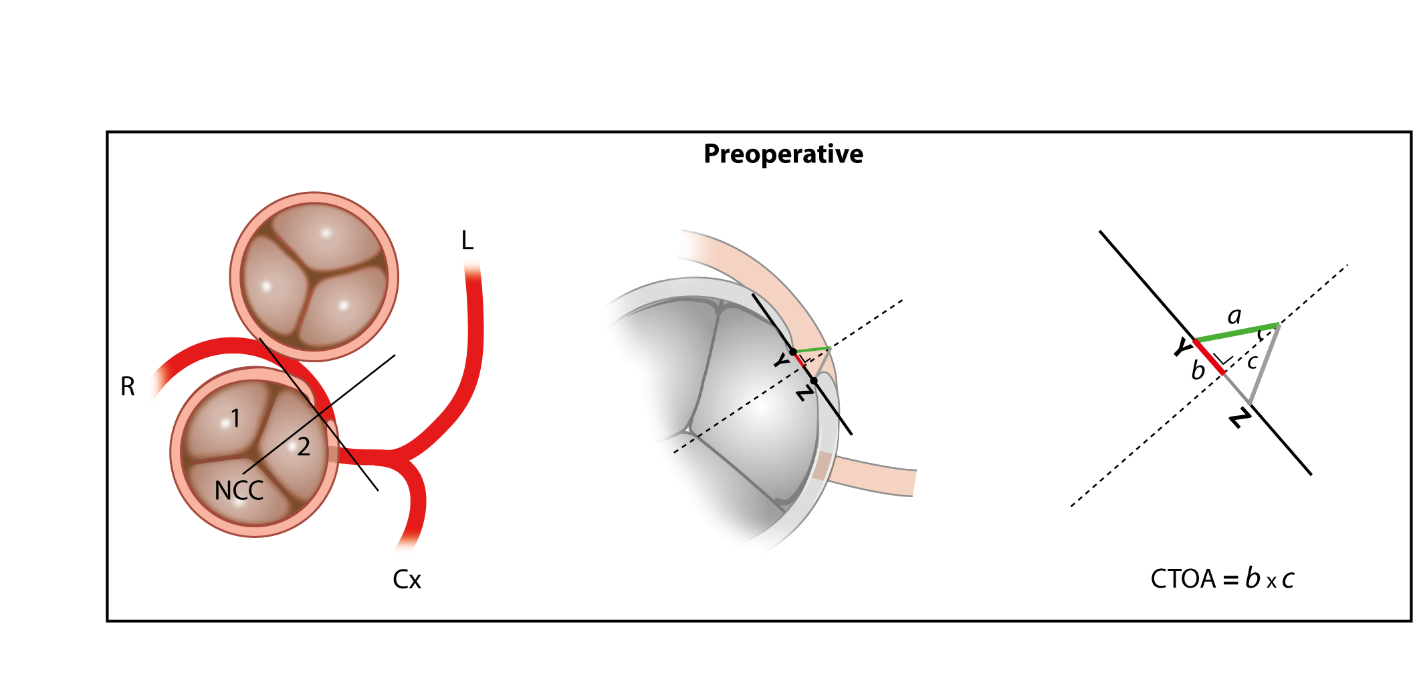

Supplement: Supplementary Figure 5 — Step 5–7. [file Image_5.TIF]
